# A model for reconstructing trends and distribution in age at first sex from multiple household surveys with reporting biases

**DOI:** 10.1016/j.epidem.2022.100593

**Published:** 2022-09

**Authors:** Van Kính Nguyen, Jeffrey W. Eaton

**Affiliations:** MRC Centre for Global Infectious Disease Analysis, School of Public Health, Imperial College London, London, United Kingdom

**Keywords:** Age at first sex, Log-skew-logistic, Age at report bias, Sexual debut, Survival model

## Abstract

Age at first sex (AFS) is a key indicator for monitoring sexual behaviour risk for HIV and other sexually transmitted infections. Reporting of AFS data, however, suffers social-desirability and recall biases which obscure AFS trends and inferences from the data. We illustrated AFS reporting biases using data from nationally-representative Demographic and Health Surveys conducted between 1992 and 2019 in Ethiopia, Guinea, Senegal, and Zambia. Based on this, we proposed a time-to-event, interval censored model for the distribution of AFS that uses overlapping reports by the same birth cohort in successive surveys to adjust for reporting biases. The three-parameter log-skew-logistic distribution described the asymmetric and nonmonotonic hazard exhibited by empirical AFS data. In cross-validation analysis, incorporating a term for reporting bias as a function of age at report improved model predictions for the trend in AFS over birth cohorts. In the four example applications, the quartiles of the AFS distribution were 16–23 years for Ethiopian and Senegalese women and 15–20 years for Guinean and Zambian men. Median AFS increased by around one to 1.5 years between the 1960 and 1989 birth cohorts for all four datasets. During adolescent and young adult ages, men tended to report an earlier AFS while women tended to report an older AFS than when asked in their late twenties. Above age 30, both male and female respondents tended to report older AFS compared to when surveyed in their late twenties. Simulations validated that the model recovered the trend in AFS in the presence of reporting biases. When there were biases, at least three surveys were needed to obtain reliable estimate for a 20-year trend. Mis-specified reference age at which AFS reporting is assumed unbiased did not affect the trend estimate but resulted in biased median AFS in the most recent birth cohorts.

## Highlights

• The log-skew-logistic distribution describes the distribution of age at first sex.
• Social desirability and recall biases can be adjusted using data from multiple surveys.
• The distribution of sexual debut affects the age pattern of population STI risk.
• Median age at first sex has increased over the past 3 decades in African populations.

## 1 Introduction

Age at first sex (AFS) refers to the age when an individual begins sexual intercourse. This indicator has been routinely collected as part of national household surveys and used for monitoring efforts for prevention of HIV and other sexual transmitted infections (STIs) as well as providing perspectives on social, gender equity, and demographic developments. Early AFS was associated with higher rates of STIs (Pettifor, 2004, Kaestle et al., 2005) while delaying AFS decreased the risk of HIV infection (Hallett et al., 2007) and contributed to curtailing HIV epidemics (Halperin et al., 2004). Early AFS could also heighten the likelihood of engaging in risky sexual behaviours that facilitate STIs (Kaplan et al., 2013).

Data on AFS are typically collected via self-report in nationally-representative household surveys. These surveys are conducted roughly every five years to track health and population indicators, such as Demographic and Health Surveys (DHS) (ICF, 2021a) or Multiple Indicator Cluster Surveys (MICS) (UNICEF, 2021). Several challenges have been identified for measuring AFS. First, monitoring AFS has focused on summary statistics such as the median AFS or the proportion sexually experienced by a particular age, for example by age 15 or age 18. These indicators do not characterise the full distribution, including tail behaviours, which inform transmission dynamics and identify subpopulations who are vulnerable to risk. Second, observations are right censored for respondents who have not had sex at the time of data collection. If not analysed appropriately, the observed median AFS is biased downwards for younger and more recent birth cohorts. A common strategy for calculating indicators is to exclude the youngest cohorts (for example respondents aged 15–19 years during the survey), but this discards information about the most recent trends, which are likely of greatest public health interest.

Third, retrospectively reported AFS collected primarily through face-to-face interviews are susceptible to recall and social desirability biases. It can be difficult to accurately recall AFS that occurred many years ago and responses have been shown to be affected by desirable standards of society, culture, and health or political campaigns (Catania, 1999, Wellings et al., 2006, United Nations, 2012). Inconsistencies in AFS reports ranged 30–56 % between surveys in the same population (Eggleston et al., 2000, Wringe et al., 2009). There was also a tendency to report older AFS over time (Zaba, 2004, Wringe et al., 2009). As the cohort aged from adolescence to adulthood, men reported higher AFS while women reported lower AFS (Mensch et al., 2003, Zaba, 2004).

In this paper, we elucidated reporting biases in empirical AFS data from multiple nationally-representative household surveys and developed and validated a survival model to estimate the distribution of AFS in a population over time, adjusting for previously described reporting biases.

## 2 Materials and methods

Our analysis consisted of four components. First, we conducted exploratory analysis of household survey data to elucidate the reporting biases described in previous literature (Zaba, 2004, Wellings et al., 2006, Wringe et al., 2009) and conceptualise bias adjustment models. Second, we empirically compared five alternative parametric survival distributions for representing the distribution of AFS. Third, we used cross-validation to compare model specifications for the survival distribution, trends by birth cohort, and bias adjustment components. Finally, we conducted simulations to assess situations where the model can reliably recover the trend in AFS and sensitivity to misspecification of model assumptions.

### 2.1 Survey data

We obtained data on AFS from multiple Demographic and Health Surveys (DHS) conducted between 1992 and 2019 in Ethiopia (female respondents; 4 surveys), Guinea (male respondents, 4 surveys), Senegal (female respondents, 8 surveys), and Zambia (male respondents, 8 surveys). Individual-level survey responses were extracted on respondents’ age, year of birth, sex, and age at first sex. AFS was reported in integer ages, which we assumed to be the completed age at which sexual debut occurred. AFS was recorded as ‘never had sex’ for some respondents, forming right-censored observations at the age at interview. Respondents who reported an unknown AFS were dropped from analysis.

### 2.2 Exploratory analysis

We explored reporting biases in AFS by comparing what the same birth cohort reported about their AFS in successive surveys. We calculated the proportion of respondents who had ever had sex before age 18 years among respondents aged 18 years and older stratified by respondents born in 1970–1974, 1975–1979, 1980–1984, and 1985–1989. Estimates for the proportion and 95 % confidence interval (CI) accounted for survey weights and clustered sampling design were calculated using the ‘survey’ package in the statistical software R (Lumley, 2004).

### 2.3 Parametric survival distributions

We considered five candidate parametric distributions to model the distribution of AFS for a birth cohort: the gamma distribution (two parameters), log-normal distribution (two parameters), log-logistic distribution (two parameters), generalised gamma distribution (three parameters), and log-skew-logistic distribution (three parameters). The log-skew-logistic distribution was derived from the skew-logistic distribution (also called generalised logistic distribution), defined by the density function

$$f\left(x\right)=\gamma p\exp\left(\mu -x\right){\left(1+\exp\left(p\cdot \left(\mu -x\right)\right)\right)}^{-\gamma -1}$$

and the corresponding cumulative distribution

$$F\left(x\right)={[1+\exp\left(p\cdot \left(\mu -x\right)\right)]\;}^{-\gamma }.$$

The parameters μ,p>0,γ>0 determine the scale, shape, and skewness of the distribution, respectively. The distribution reduces to a logistic distribution when γ=1 (Appendix - Fig. S1). The log-skew-logistic distribution was obtained by re-parameterising the scale parameter as μ=−log⁡(λ) and performing change of variable to x=log⁡(T), the log AFS. The survival function is

$$S\left(T\right)={1-\left(1+{\left(\lambda T\right)}^{-p}\right)}^{-\gamma }$$

and density function is

$$f\left(T\right)=T^{-1}\gamma p{\left(\lambda T\right)}^{-p}{\left(1+{\left(\lambda T\right)}^{-p}\right)}^{-\gamma -1}.$$

The hazard function for sexual debut at age T is

$$h\left(T\right)=-\partial \ln S\left(T\right)/\partial T=\;-T^{-1}\gamma p{\left(\left(1+{\left(\lambda T\right)}^{p}\right){\left(({1+\left(\lambda T\right)}^{-p}\right)}^{-\gamma })-1\right)}^{-1}$$

and the median AFS is $\lambda^{-1}{\left(2^{\gamma^{-1}}-1\right)}^{-p^{-1}}$.

To assess the suitability of the five distributions to model AFS distribution, we estimated the empirical smoothed hazard function and Kaplan-Meier estimator of the survival function for the pooled survey datasets from each of the four countries. The smoothed hazard function was estimated using kernel-based methods with local bandwidth selection (Muller and Wang, 1994). Parameters were estimated by maximum likelihood for each of the five parametric survival models. The resulting hazard and survival function were compared visually to the corresponding non-parametric estimates.

### 2.4 Survival model for time to AFS

AFS data were recorded as two variables: a binary indicator of whether an individual had ever had sex and, if so, an integer variable indicating at what age first sex occurred. Let $T_{i},i=1,\ldots ,n$ the AFS of individual i, and $d_{i}$ an indicator for whether the respondent reported ever having had sex such that $d_{i}=1$ indicated a reported AFS; those who answered that they had never had sexual intercourse were assumed right censored with $d_{i}=0$. For censored cases, $T_{i}$ was specified as the age at the time of interview.

We assumed that AFS was reported as the completed years at last birthday when first intercourse occurred. Thus, reported AFS was not interpreted as an exact age, but as an interval censored observation surrounding the true AFS $T_{i}^{*}$, that is, $P(T_{i}\leq T_{i}^{*}\leq T_{i}+1)$. The probability of AFS observations can be described by an interval censored survival model with the likelihood

$$L\left(\theta |,T,d\right)\propto \prod_{i:d_{i}=1}^{}w_{i}^{*}[S\left(T_{i}\right)-S(T_{i}+1)]\prod_{i:d_{i}=0}^{}w_{i}^{*}S\left(T_{i}\right),$$

in which $S\left(.\right)$ is the survival function of a selected distribution for AFS. The weights $w_{i}^{*}$ reflect unequal sampling weights determined by the survey sampling design. For each survey k, the raw weight $w_{\mathrm{ik}}$ for observation i was scaled as $w_{ik}^{*}=w_{ik}n_{\mathrm{eff}}/\sum_{i}w_{ik}.n_{\mathrm{eff}}={\left(\sum w_{ik}\right)}^{2}/\sum w_{ik}^{2}$ is Kish’s effective sample size (Kish, 1995), which accounted for the loss of sampling efficiency due to heterogeneous sampling weights.

### 2.5 Model specification for time trends and reporting biases

The five distributions considered are each characterised by a scale parameter that controls how the distribution spreads or concentrates. Depending on the distribution, other parameters including shape and skewness give finer control of the distribution’s shape and behaviours of the tails. The effects of covariates, including age and birth cohort, were modelled on the distribution’s scale parameter. We estimated

$$\mathrm{AFS}\;\sim \;\mathrm{Distribution}\left(s\mathrm{cale},\;\mathrm{shape},\;\mathrm{skewness}\right),\mathrm{scale}=\exp\left[\beta_{0}+\;f\left(\mathrm{bch}\right)\;+b\left(\mathrm{age}\right)\right].$$

The parameter $\beta_{0}$ is the intercept. The term f(bch) denotes changes in the distribution’s scale parameter over birth cohorts. This term captures changes in the true AFS over time, which may occur due to changing culture and social norms, response to public health campaigns, short-term shocks such as famine or unrest, or other factors. The term b(age) represents the bias in the scale introduced by the age at report. We considered that both birth cohort and age at report effects could be linear or nonlinear. Nonlinear effects were specified with a first-order random walk (RW1) structure. The standard normal distribution was used as prior for the intercept and linear coefficients of birth cohort and age at report. The prior distribution for the random walk precision parameters was specified using the penalised-complexity prior (Simpson et al., 2017). Sum-to-zero constraints were imposed on each of the random walk components, that is, $\sum_{age}b\left(age\right)=\sum_{yob}f\left(bch\right)=0$. The reference age for the reporting bias term b(age), at which reporting was assumed to be accurate, was age 23.

### 2.6 Model implementation and comparison

The model was implemented with Template Model Builder (Kristensen et al., 2016). Posterior distributions for model parameters were estimated in an empirical Bayesian framework using Laplace approximation for the marginal likelihood. Ten-fold cross-validation was conducted to compare distributions and model structures in real survey datasets. The data were randomly divided into ten subsets; the model was fitted with one of the subsets omitted each time and the resulting model was used to compute the out-of-sample expected log-likelihood of the omitted subset. The expected log pointwise predictive density (ELPD), defined as (Vehtari, et al., 2017)

$$ELPD=\sum_{k}\sum_{i\notin k}\;\log\left(\frac{1}{S}\sum_{s=1}^{S}p\;(y_{i\notin k}\left|\theta^{k,s}\right)p(\theta^{k,s}\left|y_{i\in k}\right)\right),$$

was reported where S=1000 is the number of draws from the parameter’s posterior distribution estimated from all partitions excluding partition k,k=1,…,10. Larger ELPD indicated better model predictions. Code is available at https://github.com/kklot/dbmethod.

### 2.7 Simulation study

To characterise the model’s ability to capture true AFS, simulated datasets were used to reflect alternative specifications about biases, trends, and the number of available surveys. We challenged the model to recover AFS of birth cohorts spanning from 1985 to 2005 using data comprised of 2, 3, 4, or up to 5 surveys. Simulated surveys were assumed to be conducted between 2001 and 2020. The steps to simulate the survey data were: (i) generate a pooled population of 1 million with year of birth assigned uniformly between 1940 and 2005, (ii) assign a true AFS distribution using the log-skew-logistic distribution for each birth cohort; the parameters were chosen such that the median AFS increased from 15 to 19, decreased from 19 to 15, or constant at 17 from 1940 to 2005, (iii) pick k=2,..,5 surveys conducted five years apart, started from 2020 and went backward in time. This ensured the target birth cohorts, 1985–2005, were fully covered in one survey and partially in the others with progressively lesser information about the recent birth cohorts. In each survey, 1000 adults aged 15–49 at the time of survey were sampled with probability of selection proportionate to the age structure of a typical African population (Appendix - Fig. S2), (iv) add a reporting bias to the true median AFS in step (ii); the bias pattern was a function of age of the respondent at the time of the survey and varied depending on the scenarios under assessment. The reported AFS for each individual was then sampled from the biased parameters.

Each survey scenario was simulated 1000 times; each time, we estimated the model parameters and reported the average difference from the true median AFS based on 1000 posterior samples. To measure if the simulated trend was captured, the absolute difference between the estimated median AFS for the 1985 and 2005 birth cohort was reported. We also reported the error in the estimated median AFS of the most recent birth cohort for its pertinent in AFS communication.

### 2.8 Ethics statement

The study involved secondary analysis of anonymised publicly available data from the Demographic and Health Survey programme following project approval (https://dhsprogram.com/data/available-datasets.cfm). Primary survey protocols were reviewed and approved by the Institutional Review Board of ICF International and the relevant national ethics committee in each country (https://dhsprogram.com/methodology/Protecting-the-Privacy-of-DHS-Survey-Respondents.cfm).

## 3 Results

### 3.1 Within-cohort inconsistency in reported AFS

**Fig. 1:**
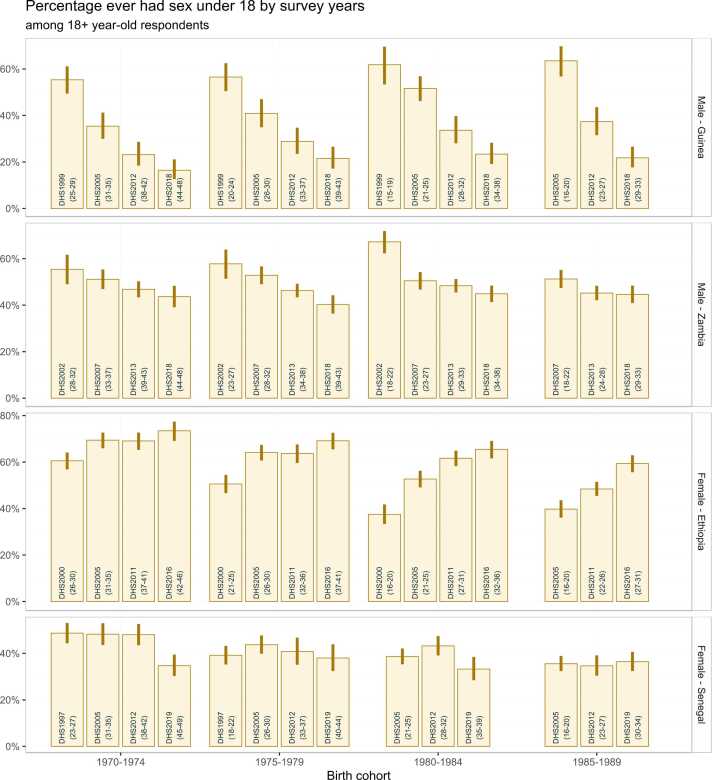
Within-cohort inconsistency in reported AFS over survey years across countries. The column’s label indicates the survey year and age at report in the bracket. Bars present point estimate and lines range are 95 % confidence interval. The presented Senegal’s surveys are selected equally distant from each other started from the earliest survey.

Fig. 1 shows the crude percentage who reported ever having had sex before age 18 years by birth cohort in successive surveys among respondents aged 18 and above. In the four countries, the percentage who reported sexual debut before age 18 varied by up to 10–20 % for the same cohort in successive surveys, indicating inconsistent reporting within the cohort. For men in both Guinea and Zambia, the proportion who reported sex before 18 reduced steadily as the cohorts aged, indicating that either younger men systematically over-reported their sexual activity or older men underreported. In Zambia the reduction was over 30 % from the first survey in 1999 to the most recent in 2018. For women in Ethiopia, the percentage within each cohort reporting sex before age 18 consistently increased in successive surveys, contrasting the pattern among men. The largest increase was as women aged from their early 20s to early 30s, with smaller changes thereafter. This pattern was consistent with the youngest women under-reporting sexual activity. In Senegal, there was some evidence that the proportion reporting AFS before 18 increased as cohorts aged through their 20s, but changes were smaller and less consistent than that of women in Ethiopia.

### 3.2 Parametric distribution for AFS

**Fig. 2:**
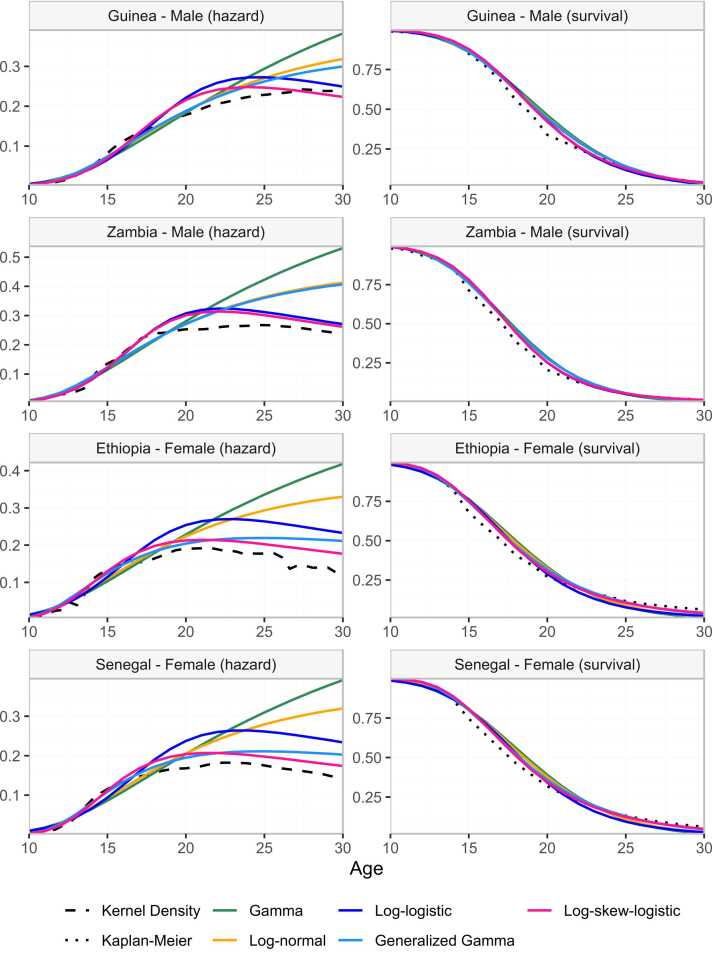
Fitted non-parametric (kernel density) and intercept-only model of parametric hazard functions of the common survival distributions to AFS data. The corresponding estimates of the survival function are shown on the right panel.

The empirical hazard rate of sexual debut increased slowly from age 10–13, then more rapidly to age 18–20, and stabilised between 0.2 and 0.3 per year (Fig. 2–dashed black line). Above age 25, the empirical hazard declined slightly for women, but by this age above 85–90 % had sexual debut. The Gompertz and Weibull distributions were not considered here because both imply an exponentially increasing hazard function, which was not suitable for these data. The best-fitting gamma and log-normal distribution did not reflect the flattening and decline of sexual debut hazard above age 20 for both males and females and the best fitting generalised gamma distribution did not capture the flattening hazard among males (Fig. 2). The log-logistic distribution and log skew-logistic were more consistent with the empirical hazard for AFS, though they did not fully capture the decline. We limited further assessments to the log-logistic and the log-skew-logistic distribution.

### 3.3 Comparison of modelled survival distributions and structures

In 10-folds cross-validation, the best model, measured by the model with the highest ELPD, was Model 5 using the log-skew-logistic distribution (Table 1). Model 5 included a smoothed term (RW1) for both the birth cohort trend in AFS and the bias in reported AFS by age at report. The log-skew-logistic distribution provided better predictions than the log-logistic distribution for all four datasets and all model specifications. The difference was largest for the Senegal and Ethiopia female datasets, for which including skewness allowed lower sexual debut rates in very young ages followed by a more rapid increase to the peak of the distribution. Introducing the age at report bias term increases the model’s predictive value substantially. The nonlinear RW1 bias term was preferred to a linear age at report effect.

**Table 1:** Ten-folds cross-validation of the log-logistic and its skewness extension version^a^.

	Model	Log-logistic	Log-skew-logistic
Ethiopia Female (n = 59,710)	M1: Intercept	-4352.4 (607.7)	-3187.8 (601.84)
	M2: + Birth cohort linear	-1808.8 (636.0)	-793.60 (622.47)
	M3: + Birth cohort RW1	-1658.6 (603.0)	-630.22 (623.47)
	M4: + Birth cohort RW1 + age at report linear	-1235.6 (625.0)	-273.69 (614.38)
	M5: + Birth cohort RW1 + age at report RW1	-931.0 (637.9)	0 (0)
Guinea Male (n = 12,936)	M1: Intercept	-721.46 (197.13)	-674.5 (199.67)
	M2: + Birth cohort linear	-539.84 (194.07)	-505.13 (194.91)
	M3: + Birth cohort RW1	-451.07 (196.39)	-416.16 (196.95)
	M4: + Birth cohort RW1 + age at report linear	-28.39 (193.14)	-5.64 (193.14)
	M5: + Birth cohort RW1 + age at report RW1	-21.77 (193.63)	0 (0)
Senegal Female (n = 110,657)	M1: Intercept	-5120.92 (581.69)	-2073.61 (589.81)
	M2: + Birth cohort linear	-3196.61 (598.73)	-418.39 (605.22)
	M3: + Birth cohort RW1	-3033.72 (608.66)	-279.88 (596.65)
	M4: + Birth cohort RW1 + age at report linear	-3036.15 (609.98)	-277.38 (600.58)
	M5: + Birth cohort RW1 + age at report RW1	-2713.72 (610.29)	0 (0)
Zambia Male (n = 37,001)	M1: Intercept	-441.97 (319.10)	-412.21 (319.71)
	M2: + Birth cohort linear	-424.85 (317.90)	-393.88 (317.57)
	M3: + Birth cohort RW1	-404.01 (318.15)	-375.63 (318.7)
	M4: + Birth cohort RW1 + age at report linear	-46.84 (316.18)	-1.11 (316.96)
	M5: + Birth cohort RW1 + age at report RW1	-44.82 (315.45)	0 (0)

a The out-of-sample expected log pointwise predictive density (ELPD) is computed for 1000 draws from the parameters posterior distributions. The values presented are the difference to the best model which is showed as zero EPLD in each country and weighting version. The plus sign preceeding the term denotes a model with intercept and the term, two pluses sign (++) denotes model include intercept, RW1 effect of birth cohort (BCH), and the effect of age at report (AAR) whether as fixed effect of as RW1 random effect.

### 3.4 Estimates of AFS trend and bias pattern

**Fig. 3:**
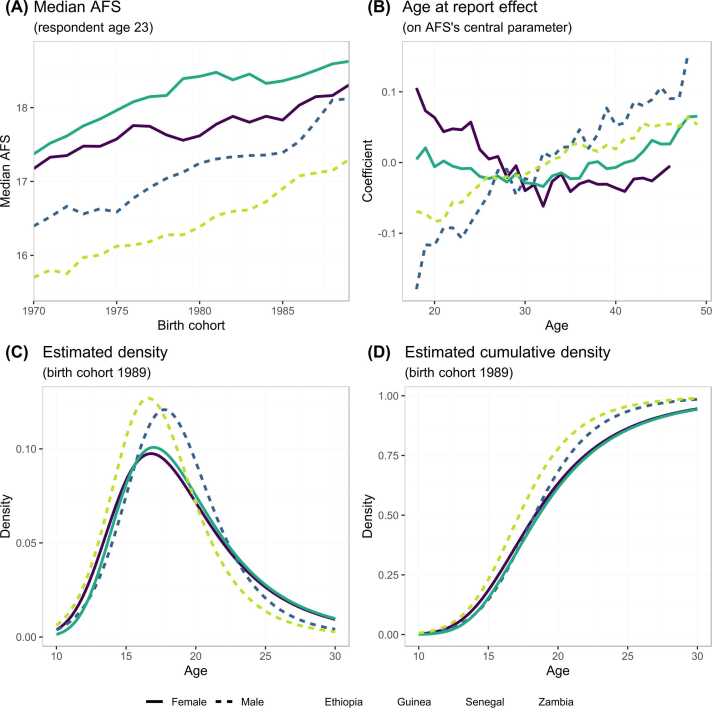
Estimated median AFS, log-skew-logistic density distribution, and age at report effect from the best model based on ELPD (Table 1). Age at report effect is fixed within birth cohort and directly change the scale parameter on the log-skew-logistic distribution.

Fig. 3 shows results of Model 5 applied to the four DHS datasets. Results were centred at the reporting age of 23 years, which is around the median age at first marriage for men (23–24 years) and above median age at first marriage for women (16–20 years) in these countries (ICF, 2021b).

Among the four datasets analysed, the estimated median AFS was highest for Senegalese women and lowest for Zambian men. Median AFS increased by around one to 1.5 years between the 1960 and 1989 birth cohorts (Fig. 3A). For the 1989 birth cohort, the rate of sexual debut peaked around age 16 or 17 (Fig. 3C). The quartiles of the AFS were 16–23 years for Ethiopian and Senegalese women and 15–20 years for Guinean and Zambian men (Fig. 3D).

**Fig. 4:**
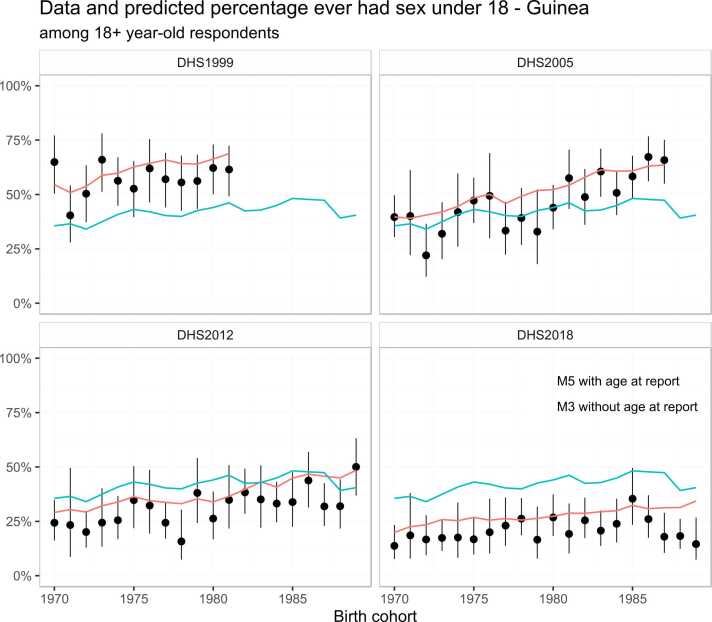
Illustration of predicted values of the model with and without taking account the age at report effect among male respondents in four DHS surveys in Guinea. Model M3 includes a RW1 term for the birth cohort but with the age at report effect ignored, model M5 is model M3 plus a RW1 model of the age at report.

The pattern of age at report bias on the reported AFS varied by sex for young adults (Fig. 3B). Consistent with the descriptive results in Fig. 1, at younger ages, male respondents tended to report a younger AFS while female respondents tended to report an older AFS than when asked in later surveys. Above age 30, both male and female respondents tended to report older AFS compared to when surveyed in their late twenties, indicated by the increasing trend in the age at report bias term above age 30 (Fig. 3B). Fig. 4 illustrates the effect of adjusting for age at report on the reported percentage ever had sex by age 18 across the four surveys conducted in Guinea; the predicted values showed that the shifts in the AFS data observed across the four surveys can be explained by the difference in respondents’ age at report.

### 3.5 Sensitivity of model to survey settings and biases

**Fig. 5:**
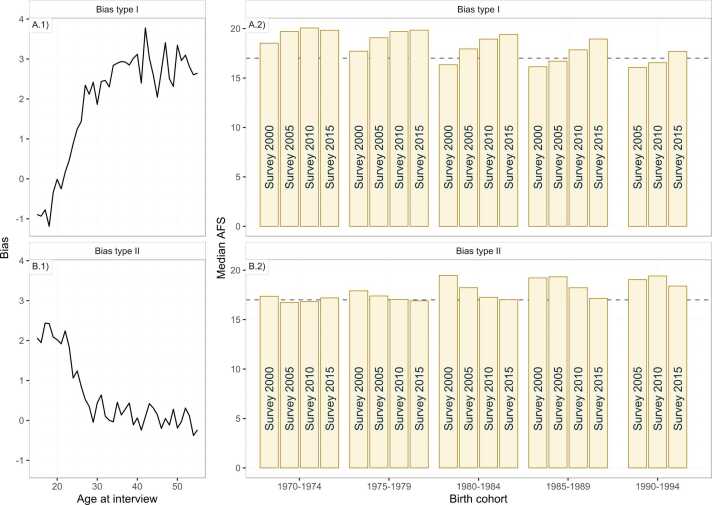
Simulated survey datasets of AFS from two patterns of AFS reporting bias as a function of age at interview. (A) Respondents under 23 years old tend to report biased earlier AFS and older respondents recall biased older AFS (Bias type I). (B) Respondents under age 30 tend to report an upward biased AFS and older respondents report unbiased AFS (Bias type II). Panels (A.1) and (B.1) show the bias on the log-scale parameter. Panels (A.2) and (B.1) show the reported median AFS by respondents in each birth cohort in successive surveys five years apart. The horizontal dashed line is the true AFS used in the simulations.

In our simulation, in addition to scenarios without reporting biases, we considered two bias patterns: a bias towards misreporting an earlier AFS by young adults as expected by young men reporting earlier sexual experience (Fig. 5A.1) and bias of younger adults reporting an older AFS consistent young women underreporting early sexual activity (Fig. 5B.1). Fig. 5 illustrates changes in median AFS that would be reported by each cohort in four surveys 5-years apart under each bias pattern when there was no change in AFS over time.

**Fig. 6:**
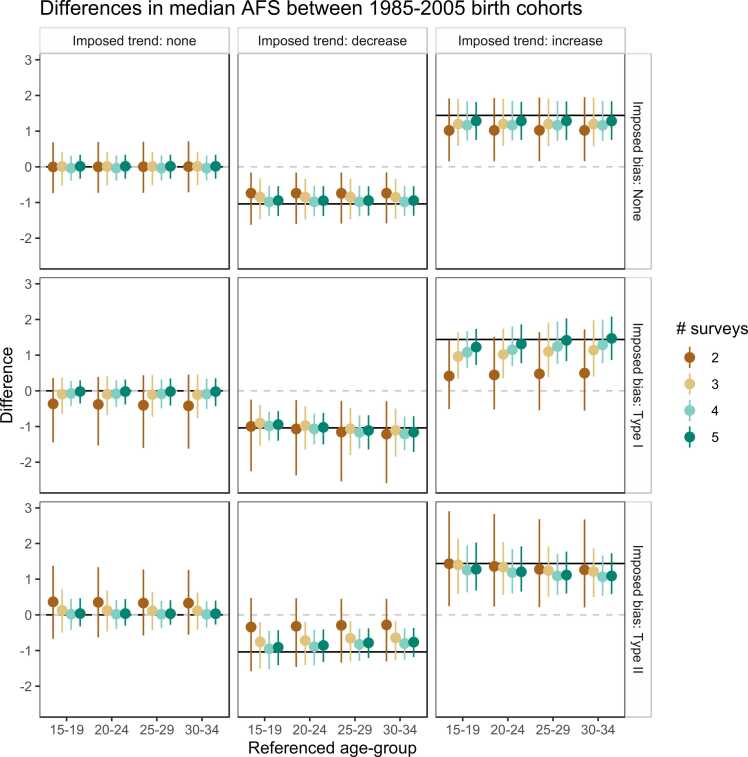
Estimated level of changes in the median AFS between the 2005 and 1985 birth cohort. The solid lines are the true difference imposed in the simulation. The types of bias are showed in Fig. 5**.** The age’s effect reference group in the x-axis illustrates the effect on the difference when the average age coefficients of ages in the group was used in generating the AFS distribution.

Fig. 6 shows the change in the median AFS between the 1985 and 2005 birth cohorts for scenarios differing in the number of surveys, trends, and bias patterns. The model identified the existence of a trend across the scenarios, including when the data were biased or the age without bias was mis-specified. When three or more surveys were available, the average percentage of simulations that correctly identified changes in median AFS of more than one-half year ranged 67–99 % (Appendix - Fig. S3). Using only the two most recent surveys was prone to incorrectly estimate the trend. For example, when there was reporting bias and no trend, estimates from two surveys suggested a trend of less than a year (Fig. 6) and only correctly identified the decrease trend 34 % of cases when data had the bias of young respondents reporting older AFS. With 4 or 5 surveys (providing more information for the older birth cohorts) the percentage increased to 85–99 %. The model was sensitive to detect trends but underestimated the true magnitude of change when fewer surveys were available. Little was gained from increasing the number of surveys from 4 to 5. Fig. 6 shows that mis-specifying the reference age-group at which reporting was assumed to be unbiased did not affect the trend estimate, but it gave incorrect estimates for the true median AFS when there were biases (Appendix - Fig. S4). The figure also shows that the magnitude and age pattern of biases were recovered correctly.

### 3.6 Implications of the model for estimated AFS distribution

**Fig. 7:**
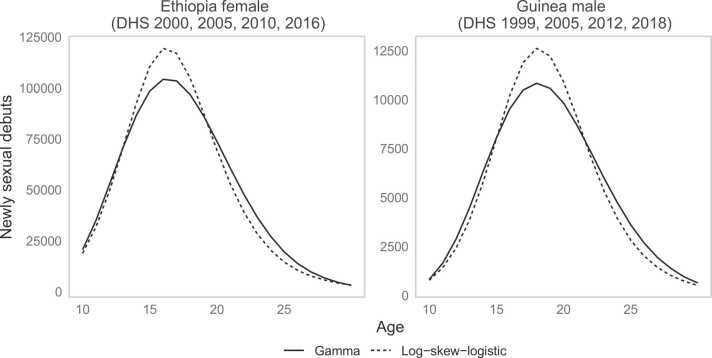
Difference in the estimated number of individuals entering sexually active population in 2019 using when assuming the log-skew-logistic distribution (dashed line) or the gamma distribution (solid line). The population size by age is taken from the World Population Prospects 2019.

Fig. 7 illustrates the differences between our bias-adjusted survival model results compared to common approaches in analysing survey AFS. The log-skew-logistic distribution, which allowed skewness and more flexible tail behaviour of the sexual debut hazard above age 20, led to differences in the estimated number of individuals who would enter the population at risk of STIs. In Guinea and Ethiopia, respectively, the number of 15–19 year olds entering the sexually active population was more than 5000 and 50,000 greater when using the log-skew-logistic distribution compared to the gamma distribution.

## 4 Discussion

Across many settings, changes in age at first sex (AFS) have been key determinants of changes in adolescent pregnancy and sexually transmitted disease, especially HIV/AIDS. However, measuring changes in this indicator over time is challenging due to biases arising from societal, religious, and cultural norms on sexual behaviours. In this study, we elucidated cases of potential social-desirability and recall biases in population survey data of AFS and developed a model mechanism to reproduce them in analyses.

In the example datasets, large within-cohort inconsistencies across surveys four to six years apart implied different patterns of sexual behaviour reporting from one survey to the next. The differences were less pronounced between surveys conducted annually in Senegal. Analyses of survey data conducted every two years also found small to no within-cohort differences (Wringe et al., 2009). The varying within-cohort inconsistency across countries and sexes suggested that bias pattern is sex- and area-specific. This might be attributed to differences in social and cultural norms regarding desired sexual behaviours. Models attempting to capture this effect will need to permit sex and country-level variations of the within-cohort inconsistency.

At each survey year, age eligibility restricts the coverage of the birth cohorts included in the sample. For example, a survey conducted in year 2020 will sample the 2000–2005 birth cohorts as they progress through sexual debut. Statistics on age at first sex indicators must be computed accounting for censoring of the AFS data, with various approaches including restricting analysis to cohorts that have largely completed sexual, obtaining median estimate from a Kaplan-Meier estimator, or modelling the data in a survival framework.

Previous studies have applied survival modelling framework in estimating AFS using the Gamma and generalised Gamma distribution (Zaba, 2004, Fagbamigbe and Idemudia, 2017) but have not considered the asymmetric hazard of sexual debut distribution. In all of our example datasets, the empirical estimate of the sexual debut hazard declined with age above age 20, which ruled out many distributions that did not permit this pattern. Cross-validation results showed that the asymmetric log-skew-logistic distribution reproduced empirical AFS data better than other commonly used distributions. Not considering the tail and skewness behaviours resulted in underestimating the peakedness of adolescents and young adults entering sexually active population. Further modelling should assess the effect of different AFS distribution assumptions on epidemic model predictions and age pattern of transmission.

Literature has identified many other factors associated with AFS such as place of residence (Janis et al., 2019), education (Hargreaves et al., 2012), ethnicity (Kaplan et al., 2013), and religious belief (Rostosky et al., 2004). While these factors could explain differences between surveys, they represent subgroup differences compared to the systematic inconsistency arising from inherent age differences between survey rounds (Eggleston et al., 2000, Zaba, 2004). Leveraging these age differences to model the biases proved to be beneficial in improving the model’s predictive ability, as demonstrated in our cross-validation results. Furthermore, the estimated bias pattern from the example data sets were consistent with bias mechanisms theorised in literature.

Our approach to accounting for reporting biases involved statistical adjustments to data reported in face-to-face interviews. Innovative survey methods that anonymise reporting to reduce social desirability biases, such as self-interview, computer-assisted self-interview, informal confidential voting interview, have been demonstrated to improve reporting of sensitive behaviours (Turner et al., 1998, Coxon, 1999, Gregson et al., 2004). Event history calendar methods have been applied to improve retrospective reporting of event dates, reducing recall biases (Glasner and van der Vaart, 2009, Kabiru et al., 2010, Luke et al., 2011, Goldberg, 2013). These tools are not used for reporting of AFS in DHS and similar health surveys. Their application could partially reduce the reporting biases identified here, reducing the need for post-hoc bias adjustments, though likely not eliminating it.

Simulation results validated that the age at report bias pattern can be recovered across the bias types and data trends (Appendix - Fig. S4), and by using three or more surveys, the trend recovered was also more likely correct (Fig. 6). As countries are progressing in monitoring and conducting more national surveys, there will be few countries with only one or two surveys in the future. As of 2021, however, there are still several countries in sub-Saharan Africa with sparse data on the AFS, for example, Angola, Central African Republic, Comoros, Eritrea, Guinea-Bissau, Sudan, and South Sudan. In these countries where few surveys are available, simultaneously modelling the effect of age at report using a spatial model could help to guide the estimates of each country leveraging the estimated bias patterns from neighbouring countries with more data in a hierarchical framework.

Simulation also showed that misspecification of the reference age at which AFS reporting is unbiased produced correct estimates of the overall trend but biased estimates of the median AFS. It is difficult to ascertain the correct reference age, which might also vary between countries. We chose the age 23 years as the reference age based on several considerations. Selecting an age at which most have had sexual debut and reached life stages in which sexual activity is normative, such as after median age at marriage, may reduce social-desirability bias. But we avoided ages many years above the median AFS to reduce recall bias for events occurring many years earlier. Furthermore, we avoided ages that are known to have other biases such as digit preferences (Camarda et al., 2008).

There are several limitations in this study. First, as we limited the assessments to the age at report bias mechanism informed by previous empirical research (Zaba et al., 2004), there are other types of biases which were not considered, for example temporal trends in reporting biases across all age groups. Second, we considered several commonly used distributions in survival analyses but might unknowingly leave out distributions with similar forms of hazard or consider semi- or non-parametric hazard models. A parsimonious parametric hazard model is convenient for regression analyses or as inputs to more complex models such as demographic or infectious disease models. Third, while the AFS distribution’s location parameter was allowed to vary over time, the skewness and shape parameter were fixed to borrow information from birth cohorts with a more complete AFS profile for the censored birth cohorts.

In conclusion, the distribution of age at first sex is asymmetric, and its hazard is nonmonotonic which can be described with the log-skew-logistic distribution. Estimating AFS trends and magnitude should account for potential biases according to the studied context. Age at report bias could mislead the current estimates of the AFS, however modelling data from several repeated surveys can help to identify the true underlying trend.

## CRediT authorship contribution statement

VKN and JWE conceived the work. VKN and JWE designed the work. VKN implemented the models. Both authors critically reviewed model results throughout the model development process. VKN wrote the first draft of the manuscript. Both authors critically edited the manuscript for intellectual content.

## Declaration of interests

JWE reports grants from Bill and Melinda Gates Foundation and NIH during the conduct of the study; grants from NIH, UNAIDS, Bill and Melinda Gates Foundation and WHO and personal fees from WHO and Oxford Policy Management outside the submitted work. KN declares no competing interests.

## Data Availability

The data can be requested at dhsprogram.com/data/Dataset-Types.cfm.
