# Supplementary material for: A model for reconstructing trends and distribution in age at first sex from multiple household surveys with reporting biases
Source: Epidemics. 2022 Sep;40:100593. doi: 10.1016/j.epidem.2022.100593 (PMC9469639; doi:10.1016/j.epidem.2022.100593)
Supplement: Supplementary file 1 — Supplementary material [file mmc1.docx]

# Appendix

**Appendix - Figure S1.** Illustration of log skew-logistic distribution with different levels of skewness in the data.

**Appendix - Figure S2.** Population age distribution used to generate simulated survey data sets reflecting more younger respondents than older respondents. Distribution derived from female respondents to the Eswatini 2007 DHS as a reference distribution typical of surveyed respondent populations in sub-Saharan Africa.

**Appendix - Figure S3.** Percentage of simulation correctly determined the trend imposed in the simulated dataset. The trend was determined correctly when the model detected a change of more than 6 months in median AFS when there was a trend imposed; changes in median AFS of less than 6 months in either of the directions were considered as no trend in this figure.

**Appendix - Figure S4.** Average difference in the estimated median AFS of the 2005 birth cohort. Different scenarios of the AFS trend, number of surveys, type of biases (none or a logistic function of age as shown in Figure 1). The age’s effect reference group illustrates the effect on the difference when the corresponding average age coefficients of age-group was used in generating the AFS distribution.
